# A double-blinded, placebo-controlled randomized trial evaluating the efficacy and safety of Zhigancao Tang granules for treating HFpEF: study protocol for a randomized controlled trial

**DOI:** 10.1186/s13063-021-05232-6

**Published:** 2021-04-20

**Authors:** Na Zhang, Yingli Zhao, Yu Liu, Nuo Tang, Wang Zheng, Meijiao Mao, Qingcheng Liu, Lin Shen, Bing Deng

**Affiliations:** Department of Cardiology, Longhua Hospital, Shanghai University of Traditional Chinese Medicine, Shanghai, 200032 China

**Keywords:** Traditional Chinese medicine, Zhigancao Tang, Heart failure with preserved ejection fraction, Randomized controlled trial

## Abstract

**Background:**

Heart failure with preserved ejection fraction (HFpEF) is a clinical syndrome characterized by diastolic dysfunction. Despite the increasing incidence of HFpEF, there is no available therapy that reduces the mortality rate of HFpEF. Zhigancao Tang has been used traditionally for the treatment of cardiovascular diseases in China. The use of traditional Chinese medicine (TCM) is associated with improvements in clinical syndromes and quality of life of patients. A randomized clinical trial should be conducted to provide clear evidence regarding the efficacy and safety of Zhigancao Tang granules for the treatment of HFpEF.

**Methods:**

A randomized, double-blinded, placebo-controlled clinical trial was proposed. A total of 122 patients with HFpEF will be randomly assigned to receive Zhigancao Tang granules or placebo for 12 weeks. The primary outcome measure is cardiac function. The secondary outcomes include measurement of the integral TCM syndrome score, echocardiography, 6-min walk test, N-terminal-pro hormone B-type natriuretic peptide level, atrial natriuretic peptide level, Minnesota Living with Heart Failure scale, and Lee’s scale. The outcome measures will be evaluated at baseline, 4 weeks, and 12 weeks. Adverse events will be evaluated from baseline till the 12-week follow-up period.

**Discussion:**

The results of this trial will demonstrate whether Zhigancao Tang granules are effective and safe for treating HFpEF.

**Trial registration:**

ClinicalTrials.gov NCT04317339. Registered on 23 March 2020.

**Supplementary Information:**

The online version contains supplementary material available at 10.1186/s13063-021-05232-6.

## Administrative information

The order of the items was modified to similar group items (see http://www.equator-network.org/reporting-guidelines/spirit-2013-statement-defining-standard-protocol-items-for-clinical-trials/).

**Table:** 

Title {1}	A double-blinded, placebo-controlled randomized trial evaluating the efficacy and safety of Zhigancao Tang granules for treating HFpEF associated with qi-yin deficiency:study protocol for a randomized controlled trial.
Trial registration {2a and 2b}.	ClinicalTrials.gov, NCT04317339. Registered on 23 March 2020.
Protocol version {3}	10 Jul 2020, V2.0
Funding {4}	This study was supported by the Shanghai Science and Technology Committee (No. 19401933500).
Author details {5a}	Department of Cardiology, Longhua Hospital, Shanghai University of Traditional Chinese Medicine, Shanghai 200032, China.
Name and contact information for the trial sponsor {5b}	Shanghai Science and Technology Committee, Contact number:021-23111111
Role of sponsor {5c}	The Shanghai Science and Technology Committee will provide the funding for this trial and supervise its progress.

## Background

### Introduction and rationale {6a}

Heart failure with preserved ejection fraction (HFpEF) is a clinical syndrome characterized by abnormal diastolic function caused by the impairment of left ventricular diastolic active relaxation and reduction of myocardial compliance. In recent years, the incidence of HFpEF has shown an increasing trend. Several studies have indicated that almost 50% of patients with heart failure have a preserved ejection fraction [1]. However, to date, there is no treatment option that can improve the outcomes in patients with HFpEF. This has led to difficulties in the management of chronic heart failure and public health, resulting in an increased economic burden.

The clinical manifestations of HFpEF are not specific, and its diagnosis is challenging. Typically, HFpEF is diagnosed based on symptoms such as fatigue, weakness, dyspnea, orthopnea, paroxysmal nocturnal dyspnea, and edema, as well as signs and evidence of preserved left ventricular ejection fraction (LVEF) and diastolic dysfunction [2].

In the general population, elderly women, rather than men, are at a higher risk of HFpEF [3]. Hypertension, atrial fibrillation, and obesity are commonly associated with HFpEF. Although the exact mechanism underlying HFpEF is yet to be clarified, several mechanisms have been proposed. These include structural remodeling (myocardial fibrosis and ventricular hypertrophy), inflammation, and microvascular dysfunction, leading to diastolic dysfunction owing to increased ventricular stiffness and decreased cardiac compliance [4].

In contrast to heart failure with reduced ejection fraction (HFrEF), there are a limited number of randomized controlled trials (RCTs) on patients with HFpEF. Furthermore, there is no treatment option available for HFpEF that can reduce the morbidity or mortality of patients. Current treatment strategies for HFpEF focus on symptomatic relief and screening of comorbidities. Diuretics are recommended to treat fluid overload, whereas other medications are not prescribed, as most hospitalizations and deaths are not attributed to chronic heart failure in patients with HFpEF. To improve the ability to exercise, the physical and diastolic function of patients with HFpEF and combined endurance and resistance training are recommended by the American College of Cardiology/American Heart Association and European Society Cardiology (ACC/AHA and ESC) [5].

Traditional Chinese medicine (TCM) involves multilevel and multitargeted treatment strategies with fewer side effects. Hence, it has been considered an alternative therapeutic strategy for treating heart failure [6, 7]. According to TCM theory, qi and yin deficiency are the primary syndromes in the early stage of heart failure. Zhigancao Tang is recorded in the Treatise on Exogenous Febrile Disease and has been used for the treatment of arrhythmia associated with qi and yin deficiency and various types of asthenia [8, 9]. Qi and yin deficiency are common syndromes in TCM, characterized by fatigue, shortness of breath, spontaneous perspiration or night sweating palpitation, dry mouth, and rapid pulse, which are often observed in cases of cardiovascular diseases as well [10]. Zhigancao Tang is composed of *Radix Rehmanniae Recens*, *Ramulus Cinnamomi*, *Radix Glycyrrhizae*, *Rhizoma Zingiberis Recens*, *Radix Glehniae*, *Radix Ophiopogonis*, *Fructus Cannabis*, and *Fructus Jujubae* (Table 1). In our previous trial, we confirmed that Zhigancao Tang can restore cardiac function and improve the quality of life in patients with heart failure. We intend to conduct a randomized, double-blind, placebo-controlled trial to further evaluate the efficacy and safety of Zhigancao Tang in the treatment of qi and yin deficiency-associated HFpEF. Based on the clinical findings from this study, a novel treatment option for HFpEF can be proposed.

**Table 1:** Components of Zhigancao Tang granules

Chinese name	Latin name	English name	Plant part
Zhigancao	*Radix Glycyrrhizae*	Honey-fried licorice root	Root
Shengjiang	*Rhizoma Zingiberis Recens*	Ginger	Root
Guizhi	*Ramulus Cinnamomi*	Cassia twig	Twigs
Beishashen	*Radix Glehniae*	Coastal glehnia root	Root
Shengdi	*Radix Rehmanniae Recens*	Dried rehmannia root	Root
Maimendong	*Radix Ophiopogonis*	Ophiopogon	Root
Maren	*Fructus Cannabis*	Edestan	Seeds
Dazao	*Fructus Jujubae*	Jujube	Fruits

### Objectives {7}

The aim of this RCT  is to evaluate the efficacy and safety of Zhigancao Tang granules for the treatment of HFpEF. The study hypothesizes that cardiac function can be improved significantly with Zhigancao Tang granule treatment, compared to that achieved using placebo treatment.

### Trial design {8}

This is a randomized, double-blind, placebo-controlled clinical trial. The aim of this study is to assess the safety and effectiveness of Zhigancao Tang granules in treating qi and yin deficiency-associated HFpEF.

## Methods: participants, interventions, and outcomes

### Study setting {9}

The study will be conducted at the Longhua Hospital affiliated to Shanghai University of Traditional Chinese Medicine (Xuhui District, Shanghai), a traditional Chinese medicine hospital that combines medical, teaching, and research domains. A total of 122 participants will be recruited from Longhua Hospital affiliated to the Shanghai University of Traditional Chinese Medicine and randomized into Zhigancao Tang and placebo groups. The trial was registered at ClinicalTrials.gov (NCT04317339).

#### Recruitment

Participants will be recruited from the Longhua Hospital affiliated to Shanghai University of Traditional Chinese Medicine. The patients will be informed about the objectives, approaches, potential adverse effects, and advantages of this trial. The planned enrollment period is 25 weeks.

#### Participant screening

All patients diagnosed with HFpEF will undergo laboratory blood testing before enrolment. The testing will include routine blood, urine, and liver and kidney function analyses. Electrocardiography will also be performed.

### Eligibility criteria {10}

#### Inclusion criteria

1. Compliance with the diagnostic criteria of Western medicine for HFpEF [2] and diagnostic criteria for TCM syndromes of qi-yin deficiency
2. Cardiac function classification by New York Heart Association (NYHA; grades I to III)
3. Age between 30 and 80 years
4. Those who volunteered to participate in clinical trial observation and signed the informed consent form
5. No intake of concomitant drugs during the observation period, except for those specified
6. Compliance with treatment completion and follow-up

#### Exclusion criteria

1. History of valvular heart disease, restrictive cardiomyopathy, or pericardial disease
2. Unstable decompensated heart failure after treatment
3. Presence of atrial fibrillation
4. Patients with severe lung, liver, endocrine system, and kidney dysfunction
5. Patients with cancer and other common malignant diseases that can reduce life expectancy
6. Pregnant or lactating women
7. Allergic constitution or history of allergy to common drugs
8. Patients with mental illness or poor compliance with TCM treatment

### Who will take informed consent? {26a}

The researchers will design the questions, recruit patients, and conduct the study. Patients will receive written information about the trial and decide if they would like to sign the informed consent form and participate in the study.

### Additional consent provisions for collection and use of participant data and biological specimens {26b}

We will confirm with the participants if their data may be used and request them to share data with the other relevant authorities.

## Interventions

### Explanation for the choice of comparators {6b}

The Zhigancao Tang granule placebo was selected as the comparator to demonstrate the efficacy and safety of Zhigancao Tang granules and decrease bias.

### Intervention description {11a}

#### Zhigancao Tang granule intervention

In the Zhigancao Tang group, patients will be orally administered a solution of the Zhigancao Tang granules in 50 mL of hot water twice a day for 12 weeks. The Zhigancao Tang granules were prepared from *Radix Rehmanniae Recens* (Shengdi, 15 g), *Ramulus Cinnamomi* (Guizhi), *Radix Glycyrrhizae* (Zhigancao, 12 g), *Rhizoma Zingiberis Recens* (Shengjiang, 9 g), *Radix Glehniae* (*Beishashen*, 30 g), *Radix ophiopogonis* (*Maimendong*, 12 g), *Fructus Cannabi*s (*Maren*, 9 g), and *Fructus Jujubae* (Dazao, 10 g).

#### Zhigancao Tang granule placebo

The placebo used will contain 10% Zhigancao Tang. Patients in the control group will consume the placebo at the same dose and schedule as those followed by patients in the Zhigancao Tang group. Both Zhigancao Tang granules and placebo will be supplied by the Chinese medicine pharmacy of Longhua Hospital affiliated to the Shanghai University of Traditional Chinese Medicine, with similar labels and packaging.

#### Basic treatment

If necessary, basic treatment could be administered as follows [2]:

1. Treatment of volume overload: sodium intake should be restricted to 2 g/day. Thiazide-like diuretics, loop diuretics, and aldosterone receptor antagonists should be administered based on the symptoms and body weight of the patients.
2. Treatment of coexisting conditions: patients with hypertension or kidney disease should receive angiotensin-converting enzyme inhibitors (ACEIs), angiotensin receptor blockers (ARBs), angiotensin receptor neprilysin inhibitors (ARNIs), and medical therapies. Revascularization should be considered in patients with coronary artery disease and clinical angina.

### Criteria for discontinuing or modifying allocated interventions {11b}

The criteria for discontinuation are as follows: (1) if anaphylaxis or serious adverse events occur, the test should be terminated based on the physician’s decision; (2) during the study, two consecutive laboratory tests will be compared, and the results of laboratory tests should be factored in while judging treatment efficacy and safety; (3) patients with poor compliance in the study (compliance with experimental drug < 80%), those with automatic halfway dressing change, or those administered other Chinese and Western drugs prohibited by the party; (4) cases of blinding not maintained halfway through the study for various reasons; (5) regardless of reason, unwillingness or inability of the patient to continue with the clinical study, or request of withdrawal from the study to the competent physician; (6) loss of patients owing to non-acceptance of the drug or the test, even when the study case did not explicitly propose withdrawal from the study.

### Strategies to improve adherence to interventions {11c}

The drug quantity and date of drug intake by each patient will be recorded. The patients will be required to return any unused drugs during each visit, which will be recorded by the researchers.

### Relevant concomitant care permitted or prohibited during the trial {11d}

The physician will determine the use of diuretics, beta-blockers, ACEIs, ARBs, or ARNIs based on the condition of the patients, and other traditional Chinese medicines should be forbidden.

### Provisions for post-trial care {30}

Not applicable, as there is no anticipated harm or compensation required in lieu of trial participation.

### Outcomes {12}

#### Primary outcome

- Cardiac function (from baseline to weeks 4, 12, and 24)
  - ◦ Significant effects: cardiac function restored to level 1 or increased by level 2
  - ◦ Effective: heart function improved to level 1, but not to level 2
  - ◦ Invalid: no change in cardiac function
  - ◦ Deterioration: decrease in cardiac function by a single grade or more

#### Secondary outcomes

- Echocardiography (from baseline to weeks 4 and 12): Echocardiographic measurement of LVEF, left ventricular end diastolic diameter (LVEDd), left ventricular end-systolic diameter (LVESD), ratio of left ventricular early diastolic fast filling peak to late diastolic filling peak (E/a), and ratio of early diastolic velocity of mitral valve to early diastolic annular velocity (E/e) [11].
- Lee’s scale (from baseline to weeks 4, 12, and 24)
  - ◦ Significant effect: decrease in score by > 75% after 12 weeks
  - ◦ Effective: decrease in score by 50%–75% after 12 weeks
  - ◦ Invalid: decrease in score by < 50% after 12 weeks
  - ◦ Aggravation: score is unchanged or higher than the score recorded before the treatment
- The Minnesota Living with Heart Failure Questionnaire (MLHFQ) scale (from baseline to weeks 4, 12, and 24): The symptoms of heart failure substantially affect patients, and the MLHFQ scale is used worldwide to assess the quality of life in patients with heart failure [12]. The MLHFQ scale, consisting of 21 questions related to physical and emotional changes, will be used to assess the impact of heart failure on the quality of life in the last 4 weeks. Higher scores indicate worse outcomes. Patients will be assessed based on the scale before and after the trial.
- Six-minute walking test (from baseline to weeks 4, 12, and 24): This test is a measure of the greatest distance the patient can traverse by walking on a hard ground without obstacles within 6 min at the fastest possible speed. It provides a comprehensive assessment of the exercise capacity of the patient. The differences in the distance covered by the patients in each group will be observed before and after the treatment to evaluate their exercise endurance and quality of life.
- NT-proBNP, ANP (from baseline to weeks 4 and 12): NT-proBNP is a reliable and sensitive indicator of heart failure. Its elevation is positively correlated with the severity of heart failure. Changes in the levels of NT-proBNP and ANP in each group will be measured before and after treatment.
- Integral TCM syndrome score (from baseline to weeks 4 and 12; the scores and details are presented in Tables 2 and 3): A scale of 0–6 points and half quantitative points will be used to score the patients according to the severity of clinical symptoms.
  - ◦ Significant effect: inhibition or complete suppression of primary and secondary clinical symptoms and reduction of syndrome score by ≥ 70%
  - ◦ Effective: significant improvement in clinical symptoms, and reduction of syndrome score by 30–70%
  - ◦ Ineffective: reduction of treatment syndrome score by < 30%
  - ◦ Aggravation: the score after treatment exceeds the score before treatment

**Table 2:** Traditional Chinese medicine symptom score

	None	Mild	Moderate	Severe
Palpitation	0	2	4	6
Panting	0	2	4	6
Lassitude	0	2	4	6
Spontaneous sweating	0	2	4	6
Night sweating	0	2	4	6
Dizziness	0	2	4	6
Dry mouth	0	2	4	6
Vexation	0	2	4	6

**Table 3:** Detailed description of symptoms according to traditional Chinese medicine

	None	Mild	Moderate	Severe
Palpitation	No symptom	Occasional occurrence; slight uncomfortable feeling	Regular occurrence; lasts for a long duration; intense uncomfortable feeling	Frequent occurrence; uncontrolled; considerable influence on the quality of life
Panting	No symptom	Slight and no influence on routine activities	Heavy, but can still manage to perform routine activities	Too heavy; cannot perform routine activities
Lassitude	No symptom	Slight and can work	Heavy, but can still manage to work	Too heavy; cannot work or continue routine activities
Spontaneous sweating	No symptom	Slight sweating after activities	Heavy sweating without any activity	Profuse sweating
Night sweating	No symptom	Occasional occurrence; sweating primarily in the head	Regular occurrence; sweating primarily on the chest and back	Frequent occurrence; sweating all over the body
Dizziness	No symptom	Slight, and no influence on work	Heavy, but can still manage to work	Too heavy; cannot work
Dry mouth	No symptom	Slight; no need to drink water	Severe; need to drink water occasionally	Intolerable; need to drink water frequently
Vexation	No symptom	Slight and occasional occurrence	Heavy and regular occurrence, but tolerable	Unbearable; severe impact on the quality of life

#### Safety monitoring

Routine blood and urine analyses, electrocardiography, and liver and kidney function tests will be performed before and after treatment. Adverse events and severe adverse events will be recorded at each visit.

### Participant timeline {13}

**Fig. 1:**
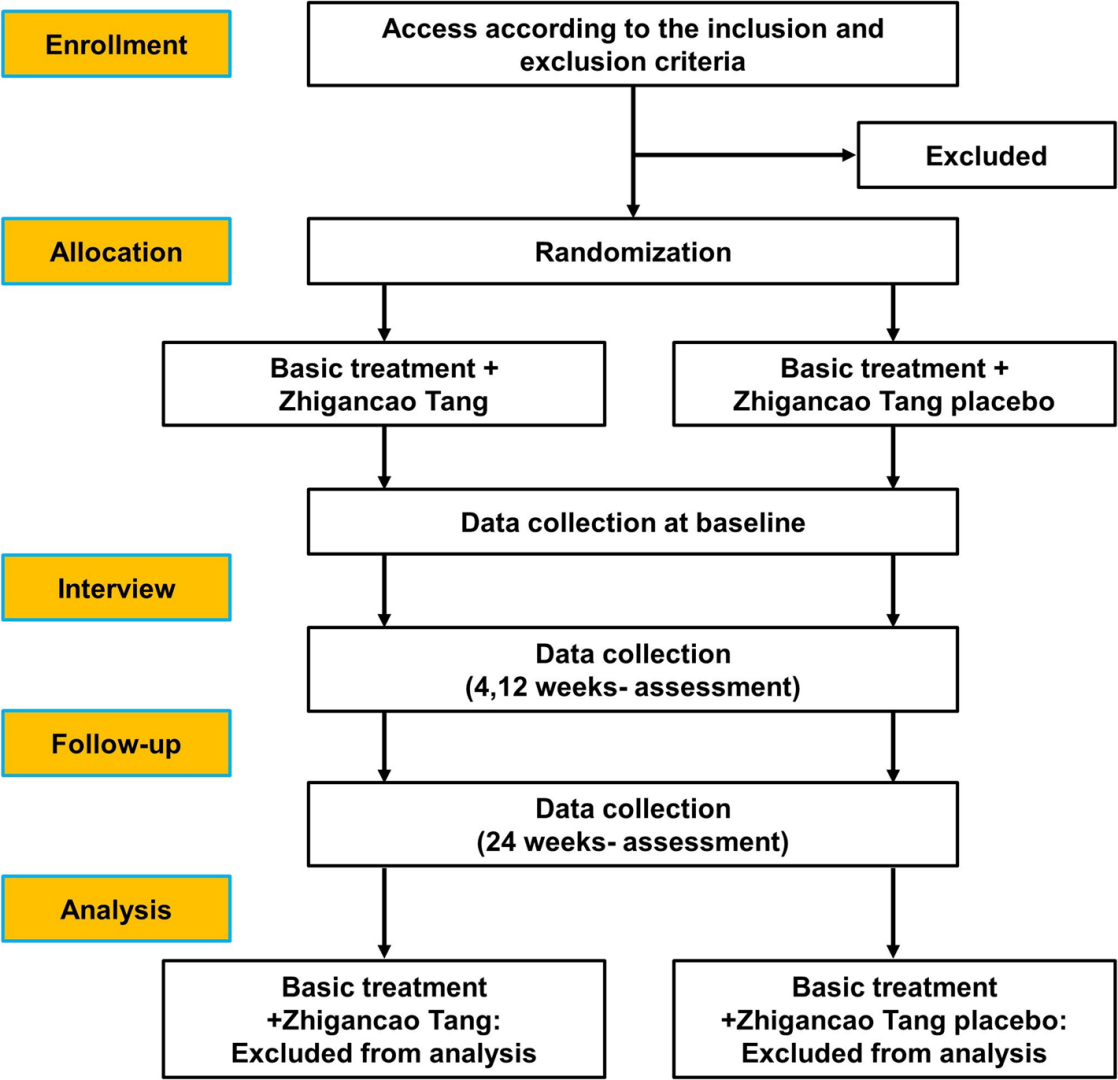
Flow diagram of the study process

Recruitment has commenced in April 2019 and will end in April 2022 (Fig. 1). The schedule for registration, intervention, and evaluation are listed in Table 4.

**Table 4:** Schedule of enrollment and assessments

Study period					
Time point	Enrolment	Allocation	Post-allocation		Follow-up
	−1 week	Week 0	Week 4	Week 12	Week 24
Enrolment					
Eligibility screen	×				
Informed consent	×				
Allocation		×			
Interventions					
Zhigancao Tang granules					
Zhigancao Tang granule placebo					
Outcome assessments					
Cardiac function		×	×	×	×
Echocardiography		×	×	×	×
Lee’s scale		×	×	×	×
MLHFQ		×	×	×	×
6MWT		×	×	×	×
NT-pro BNP, ANP		×	×	×	
ITSS		×	×	×	×
Safety assessments					
Blood routine		×		×	
Urine routine		×		×	
Liver and kidney function		×		×	
electrocardiograph		×		×	
AE		×	×	×	×
Compliance assessments			×	×	

AE, adverse events; ANP, atrial natriuretic peptide; ITSS, integral Chinese medicine syndrome score; MLHFQ, Minnesota Living with Heart Failure questionnaire scale; NT-proBNP, N-terminal pro-brain natriuretic peptides

### Sample size {14}

The sample size for this study was calculated based on the expected value of efficacy, according to the clinical trial results and analysis of data from previously published articles [13]. The total efficacy rates in the study and control groups were 93.3% and 73.3%, respectively. The significance level (alpha) was set at 0.05, and the statistical power was set at 80%. Based on our calculation, using the PASW statistical software (V.18.0), the experimental and control groups will each require a sample size of 51 patients. Considering a 20% loss to follow-up, 61 participants are required in each group. Therefore, this trial will require at least 122 participants in the current study.

### Recruitment {15}

We will use three major strategies to ensure adequate participant enrolment: (1) posting recruitment information in hospital areas, such as the outpatient hall; (2) posting recruitment information on WeChat, a popular communication platform; and (3) introduction of the trial to prospective patients by researchers during the treatment process; the researchers will ask the patients if they would like to participate.

## Assignment of interventions: allocation

### Sequence generation {16a}

The ResMan clinical trial public will be used as the central randomization tool to automatically generate random numbers. Participants will be required to register once they meet the inclusion criteria. Each participant will be provided an ID as a unique identification number and assigned to one of the two groups by logging on to the website of the randomization center.

### Concealment mechanism {16b}

The random coding table will be generated by the central random platform and sealed by the GCP specialist of Longhua Hospital and submitted to the project leader for preservation. The drugs will be packaged and confirmed as a blinded treatment by a GCP specialist at the Longhua hospital; the random codes will be mentioned on the drugs and the box label.

### Implementation {16c}

Random sequences will be generated by statisticians who will not participate in the implementation of the project. In addition, neither researchers nor patients will have access to the random sequences.

## Assignment of interventions: blinding

### Who will be blinded {17a}

All participants, subjects, and study monitors will be blinded until the trial is completed.

### Procedure for unblinding if needed {17b}

For emergency blinding, there will be an emergency envelope for every case, with information about the coding and groups. It is necessary to be aware of the drugs used by the patients in case of any serious event, and patients need to be treated. The project leader decides to initiate unblinding and open the corresponding emergency envelope. Once the emergency envelope is opened, the case is treated as a shedded case.

## Data collection and management

### Plans for assessment and collection of outcomes {18a}

In this 24-week trial, all participants will be treated with the Zhigancao Tang granules or placebo for 12 weeks, with follow-up continuing for another 12 weeks. Data will be recorded in the case report forms (CRFs) at each visit and entered in Microsoft Excel by two statisticians working independently.

### Plans to promote participant retention and complete follow-up {18b}

All researchers will be advised to treat the participants in a friendly and respectful way and be patient, answering all questions they may have while encouraging them to participate. If the patients find it difficult to reach the hospital, the round-trip fare will be reimbursed. The researchers will send reminders to the patients 2 to 3 days before each follow-up.

### Data management {19}

An electronic data collection system (EDC) was adopted for online data acquisition in this clinical trial. To ensure the safety and privacy of the original data, the research group will arrange full-time CRC to perform data entry to avoid the issues associated with data transportation. Second, medical coding will be carried out in accordance with CTCAE5.0, MedDRA-23.0, and other reference standards. Data management plans will be designed by the data administrator before the trial, which will include data management plan management, studies on the general situation, such as research purpose, research of the overall design, data management of schedules, rules and responsibilities of the relevant personnel, including the EDC system administrator (Admin), project data manager (DM), the researchers (absence), data operator, and arbitrator (CRA), among others, on design, data management, including database design and logical design verification, distribution of users and access, data processing, data quality control, closed database, data safety and security measures, and EDC system contingency plans. The data administrator will upload the random system designed by the statistician in a prescribed format for screening and randomization to be performed later.

### Confidentiality {27}

Once the recruitment commences, the patients will directly contact the researcher to avoid revealing their personal information to others. The researchers shall not discuss the patients’ condition and information in public areas, and patient data cannot be released in the media or on the internet. All documents will be stored confidentially and will only be accessible to members of the trial team. An EDC was used for online data collection in this clinical trial. We will assign specific permissions to the different groups of researchers in the project to ensure the security and confidentiality of data. All data collected in the system will be backed up with reliable data and privacy security mechanisms, and desensitization technology will be adopted to ensure absolute privacy of the data of project participants. All data collected during the study will remain strictly confidential and only be accessed by researchers; the data will be made available to other researchers upon request after the analyses and publication of primary findings. In addition to the patient’s initials, the patients’ outpatient or inpatient number will also be recorded in the CRF during the trial: “Patient initials will be recorded on the CRF cover and information page, not signatures or names.” Even if the initials are duplicated, the outpatient or inpatient number can be used as a unique ID to identify the relevant information. All original data will be recorded in the study medical records and entered in the EDC by the assigned access researcher.

### Plans for collection, laboratory evaluation, and storage of biological specimens for genetic or molecular analysis in this trial/future use {33}

Not applicable.

## Statistical methods

### Statistical methods for primary and secondary outcomes {20a}

Data analysis will be completed using the Statistical Packages of Social Sciences software (version 24.0). Qualitative indicators will be described in terms of frequency tables, percentages, or component ratios, whereas quantitative measures will be described in terms of mean, median, lower quartile (Q1), upper quartile (Q3), minimum, and maximum. For the comparative analysis of the two groups, the chi-square test, Fisher’s exact probability method, and Wilcoxon rank and inspection will be applied for qualitative data and *t* test will be applied for quantitative data, while the *t* test will be applied for quantitative data with normal distribution. Homogeneity test of variance will be conducted among the groups, with a cutoff level of 0.05, and the Satterthwaite method will be used to conduct the corrected *t* test when the variance is uneven. If the data do not follow normal distribution, the Wilcoxon rank test and inspection will be performed. The two-sided test will be uniformly used in the hypothesis test, and the test statistics and their corresponding *P* values will be provided, with *P* values ≤ 0.05 considered statistically significant and *P* values ≤ 0.01 considered highly statistically significant.

#### Missing data

Any missing data will be treated as a failure using the intention-to-treat principle, and the reason will be recorded.

### Interim analyses {21b}

We started performing statistical analysis when the samples reached 60. After the interim results are obtained, the primary investigator will determine whether the experiment will be continued.

### Methods for additional analyses (e.g., subgroup analyses) {20b}

We will perform subgroup analysis by gender or age in the future.

### Methods in analysis to handle protocol non-adherence and any statistical methods to handle missing data {20c}

We will perform per-protocol and intention-to-treat analyses. Participants with poor compliance (compliance of experimental drug < 80%) or those no longer receiving medication and undergoing testing during the study will be excluded from the per-protocol analysis. Researchers will be advised to take active measures to complete the last test if the participants drop out of the trial or are lost to follow-up for the intention-to-treat analysis. Missing data will be imputed using a multiple imputation procedure via chained equations.

### Plans to give access to the full protocol, participant level-data, and statistical code {31c}

The complete protocol, participant level data, and statistical code are available on request from the corresponding author.

## Oversight and monitoring

### Composition of the coordinating center and trial steering committee {5d}

The coordinating center is the Department of Cardiology of Longhua Hospital Affiliated to Shanghai University of Traditional Chinese Medicine, and the head of the department is the principal investigator, who will conduct the trial, identify potential recruits, obtain consent, and report to the trail steering committee. The trial steering committee is the Good Clinical Practice office of Longhua Hospital, affiliated with the Shanghai University of Traditional Chinese Medicine. The responsibilities include agreement and amends to the protocol, receiving reports from the coordinating center, supervising the trial, reviewing trial progress, and reviewing the paper for publication. Meetings are conducted every 6 months.

### Composition of the data monitoring committee, its role and reporting structure {21a}

The data monitoring committee (DMC) includes a researcher, who will not be involved in the data collection, data manager, biostatistician, and ethicist (independent from the sponsor and free from competing interests). The DMC will conduct periodic interim evaluations and discontinue the trial if there are obvious differences between the two groups.

### Adverse event reporting and harms {22}

Adverse events will be recorded and reported to the researchers, including the timing, severity and duration, actions taken, and outcomes. Zhigancao Tang granules may cause diarrhea, abdominal distension, dizziness, and anaphylaxis. The researchers will record the adverse events and decide whether to terminate the treatment based on the patient’s condition and report the same to the DMC and ethics committee. Other unintended effects of trial interventions or trial conduct will also be reported.

### Frequency and plans for auditing trial conduct {23}

The project management group will meet to review trial conduct every three months, and the trial steering group, DMC, and ethics committee will meet to review trial conduct every 6 months.

### Plans for communicating important protocol amendments to relevant parties (e.g., trial participants, ethical committees) {25}

When there are significant changes in the study process, we will notify the sponsor firstly, and then the center will be notified by the principal investigator, and an amended copy of the protocol will be sent to the investigator site file to be added. Any breach of this protocol will be fully documented using the breach report form. Then, the protocol will be updated in the clinical trial registry.

### Dissemination plans {31a}

The final data will be submitted to the Shanghai Science and Technology Committee in the form of a research report by the researchers after completion of the study. The results will be published in a peer-reviewed academic journal to share the findings with the general public and healthcare professionals.

## Discussion

HFpEF is a clinical syndrome classified as heart failure with a reduced or preserved ejection fraction. However, it remains debatable if these are two different entities or different stages of a single process [14]. The prevalence of HFpEF increases with age, and previous studies have shown that it is more common in women than in men of the same age [15–17]. The pathophysiology of HFpEF remains poorly understood and has several phenotypes, which makes its diagnosis and treatment considerably challenging [4]. HFpEF has garnered increasing attention in recent years owing to the high mortality rate reported. Furthermore, diastolic dysfunction is a hallmark of HFpEF, which is associated with adverse prognosis [18, 19]. Although there have been several large prospective RCTs such as PEP-CHF, CHARM-Preserved, I-PRESERVE, TOPCAT, Aldo-DHF, and SENIORS, therapies for reducing mortality in HFpEF are yet to be reported [20–25].

Zhigancao Tang has been used traditionally for treating arrhythmia, viral myocarditis, and ischemic cardiomyopathy [26–28]. Several clinical trials have shown that Zhigancao Tang can improve symptoms such as heart palpitations, shortness of breath, and fatigue in patients with premature ventricular contractions [9]. A case report suggested that modified Zhigancao Tang could improve exercise tolerance and alleviate pulmonary edema and cardiomegaly in an 18-year-old adolescent male with congestive heart failure induced by chemotherapy [29]. Furthermore, Zhigancao Tang has been reported as the most frequently used Chinese herbal prescription formula for treating heart failure, as it can supply Yang-Qi, nourish the Ying-blood, and strengthen the heart spirit to relieve symptoms of heart dysfunction when co-administered with standard anti-heart failure treatments [30].

Based on our analysis of literature in PubMed, Embase, Cochrane Library, China National Knowledge Infrastructure, and Clinical Trials up to April 2020, we found that no large randomized clinical trial have been conducted to evaluate the safety and efficacy of Zhigancao Tang in treating HFpEF. To the best of our knowledge, this is the first randomized, double-blind, placebo-controlled trial to investigate the effectiveness and safety of Zhigancao Tang. First, a double-blinded study and a placebo were designed to avoid bias owing to subjectivity, personal preferences, and placebo effects. Second, since HFpEF has several phenotypes, patients with atrial fibrillation were excluded to select a specific patient population. However, this study has a few limitations. This is a single-center trial conducted in China. Considering the large population size in China, this study may not adequately represent patients from other regions. Moreover, the primary outcome was set as cardiac function, which is a subjective indicator; therefore, a certain bias remains.

This randomized, double-blind, placebo-controlled trial will evaluate the clinical efficacy and safety of Zhigancao Tang granules for treating HFpEF and its efficacy in improving cardiac function, clinical symptoms, quality of life, and exercise capacity in patients with HFpEF; the trial will provide useful data for a novel strategy for HFpEF treatment.

## Trial status

The protocol version number and date were V1.0 and April 20, 2020, respectively. Patient recruitment has commenced in April 2019 and will end in April 2022.

## Supplementary Information

**Additional file 1.**

## Abbreviations

ACC/AHA: American College of Cardiology/American Heart Association
ACEI: Angiotensin-converting enzyme inhibitors
ANP: Atrial natriuretic peptide
ARB: Angiotensin receptor blocker
ARNI: Angiotensin receptor neprilysin inhibitor
CRFs: Case report forms
ESC: European Society Cardiology
GCP: Good Clinical Practice
HFpEF: Heart failure with preserved ejection fraction
LVEDD: Left ventricular end-diastolic diameter
LVEF: Left ventricular ejection fraction
LVESD: Left ventricular end-systolic diameter
MLHFQ: Minnesota Living with Heart Failure questionnaire scale
NT-proBNP: N-terminal pro-brain natriuretic peptides
NYHA: New York Heart Association
