# Supplementary material for: A double-blinded, placebo-controlled randomized trial evaluating the efficacy and safety of Zhigancao Tang granules for treating HFpEF: study protocol for a randomized controlled trial
Source: Trials. 2021 Apr 20;22:293. doi: 10.1186/s13063-021-05232-6 (PMC8056488; doi:10.1186/s13063-021-05232-6)
Supplement: Supplementary file 1 — Additional file 1. [file 13063_2021_5232_MOESM1_ESM.pdf]

学校代码：10228
学　　号：20112351

硕士学位论文

（临床医学专业学位）

活血利水法对左心室射血分数保留心衰患者心脏舒张功能的影响

THE EFFECT OF BLOOD ACTIVATING WATER RELIEVING METHOD TO CARDIAC DIASTOLIC FUNCTION IN PATIENTS WITH HF-PEF

专　　业：中西医结合临床
研 究 生：程霄瀚
导　　师：刘　莉　教授

黑龙江中医药大学

二〇一四年六月

Abstract

Objectives: To observe the clinical efficacy of blood activating water relieving method (BAWRM) in treating heart failure with preserved left ventricular ejection fraction (HF-PEF) , and the effect to left ventricular diastolic function.

Methods: Sixty patients with HF-PEF were randomly divided into control group (n=30) and treatment group (n=30). Both of groups were given conventional treatment for four weeks, while treatment group had BAWRM decoction for addition. Traditional Chinese medicine (TCM) syndrome scores, B-type natriuretic peptide (BNP), early diastolic velocity of mitral annulus(E') and ratio of the mitral inflow E wave to the tissue Doppler E' wave(E/E') were observed. The data was analyzed by SPSS 17.0 ($P<0.05$).

Results:

1. The TCM syndrome scores of treatment group are lower than control group ($P<0.05$).

2. The serum BNP levels of treatment group are lower than control group ($P<0.05$).

3. The E/E' ratio of treatment group are lower than control group ($P<0.01$).

Conclusions:

1. BAWRM was an effective way for the treatment of HF-PEF.

2. BAWRM could obviously relieve TCM syndrome of patients with HF-PEF.

3. BAWRM could significantly reduce serum BNP level and E/E' ratio, which means improving left ventricle diastolic function.

Key words: blood activating water relieving method

heart failure with preserved left ventricular ejection fraction

B-type natriuretic peptide
